# A novel Ag-loaded 4 Å zeolite as an efficient catalyst for epoxidation of styrene†

**DOI:** 10.1039/d4ra00758a

**Published:** 2024-06-19

**Authors:** Junzhong Wang, Qiancheng Zhang, Ying Li, Tong Xu, Yinghui Sun, Jie Bai

**Affiliations:** a College of Chemical Engineering, Inner Mongolia University of Technology Hohhot 010051 People's Republic of China syh9025@imut.edu.cn baijie@imut.edu.cn; b Inner Mongolia Key Laboratory of Industrial Catalysis Hohhot 010051 People's Republic of China

## Abstract

In this study, a novel Ag-loaded 4 Å zeolite was synthesized through the combined action of strong ultrasound and a high-voltage electrostatic field (the Z-Ag-UE) and its catalytic activity was evaluated in the epoxidation of styrene. The prepared catalysts were characterized using XRD, SEM, XPS, BET, TG, ICP-OES. The results showed that the silver evenly dispersed inside the octahedral 4 Å zeolite structure rather than being attached to the surface of the material like in the impregnation method, and this Ag-loaded 4 Å zeolite had a high surface area, uniform particle size distribution, and excellent high temperature thermal stability. The catalytic performance of the Ag-loaded 4 Å zeolite was investigated by varying the reaction conditions such as the amount of catalyst, temperature, and reaction time. Under optimized conditions, the Ag-loaded 4 Å zeolite showed high selectivity and conversion for the epoxidation of styrene, achieving a conversion rate of up to 98% and a selectivity of 94%. In particular, the catalyst had excellent recyclability and was reused more than fifteen times with the catalytic performance remaining unchanged. This method of loading metal prepared under external field conditions provides a new method and idea for future research in related fields.

## Abstract 2

a novel Ag-loaded 4 Å zeolite synthesized through ultrasound and electrostatic field showed high selectivity (94%) and conversion (98%) for the epoxidation of styrene and was reused more than fifteen times with the performance remaining unchanged.

## Graphical abstract

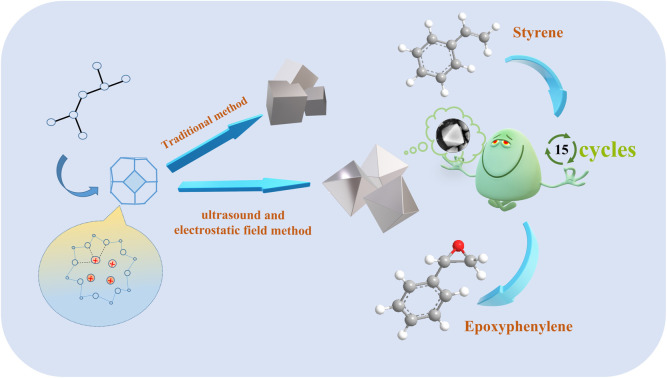

## Introduction

Styrene oxide, also known as phenyl ethylene oxide, plays a crucial role as an intermediate in organic synthesis. It is utilized in the production of various organic compounds that find applications across different industries.^1^ The main product of the styrene epoxidation reaction, styrene oxide is accompanied by by-products such as benzaldehyde, phenylacetaldehyde, and benzoic acid. However, challenges like low product selectivity and environmental concerns stemming from the generation of by-products persist in the synthesis process. Therefore, there is a pressing need to develop a more environmentally friendly and less polluting method of synthesis. Catalysts are instrumental in addressing the issues associated with the epoxidation of styrene, given the propensity for by-product formation. Due to by-products being easily produced, catalysts play a very important role in the epoxidation process of styrene. Jia *et al.*^2^ applied CuO nanorods to the epoxidation reaction of styrene and achieved relatively good catalytic effects. Li *et al.*^3^ developed a cobalt oxide composite catalyst containing copper, and obtained a series of catalysts based on different copper cobalt ratios to catalyze the epoxidation reaction of styrene with TBHP as the oxygen source. Lashanizadegan *et al.*^4^ composed a composite catalyst of Ag and CuO, and also achieved relatively good catalytic effects. Zhang *et al.*^5^ prepared a Fe_3_O_4_–CuO composite structure catalyst combined with mesoporous SiO_2_. Their synthesis method can be extended to other catalytically active metals or metal oxides, allowing them to be composited together to form a multifunctional composite nanocatalyst.

Heteroatom-substituted molecular sieve catalysts have also been used to catalyze styrene epoxidation reactions. Some researchers combined metal ions and metal complexes with zeolite to develop catalysts with excellent catalytic properties.^6,7^ Suib *et al.*^8^ developed a versatile template-free method and successfully realized general preparation of six mesoporous transition oxides such as CuO, CoO, and spinel MCo_2_O_4_ (M = Co, Cu, Mn, and Zn). In addition, the mesoporous Co_3_O_4_ was identified as surface-defect rich, which ended it with excellent catalytic activity. Ke *et al.*^9^ developed a photothermal catalytic approach with LaSrCo_*x*_Fe_2−*x*_O_6_ double perovskite materials as heterogeneous epoxidation catalysts. Highest active LaSrCo_1.6_Fe_0.4_O_6_ (LSCF-1.6) material exhibited a conversion of 100% for styrene and selectivity of 87.8% for styrene oxide under an oxygen atmosphere (bubbled with oxygen, 100 °C). Based on the analysis of the results of electron paramagnetic resonance, density functional theory calculations, and controlled experiments, it was found that the singlet oxygen was the key reactive oxygen species for epoxidation of styrene over the LSCF-1.6 catalyst. Some researchers^10–12^ used different transition metals (Fe^2+^, Co^2+^, V^2+^, Cu^2+^) to load Schiff base complexes onto graphene oxide to study the catalytic epoxidation performance of styrene and the results were good. Numerous studies have shown that the noble metal-based catalysts exhibited excellent catalytic performance.^13–15^ Nanosilver catalyst also has good antibacterial properties and catalytic activity, especially for oxidation reactions. More importantly, it has the good catalytic activity of noble metal catalysts, but is relatively cheap compared with other noble metal catalysts, making it more suitable for industrial catalysis. Although research on silver nanocatalysts is now very extensive, the research on the catalytic activity of silver nanoparticles for organic reactions is still in its infancy, and is still a relatively big challenge in current organic synthesis. In recent years, there have been increasing reports about the use of Ag-based catalysts in the epoxidation reaction of styrene.^1^ In recent research work, silver nanoparticles have been loaded onto different supports such as mesoporous silica, zeolite, carbon fibers, *etc.* to catalyze the epoxidation reaction of styrene. Researchers^16–19^ combined nanoparticles with other materials and achieved controllable synthesis of nanoparticles, nanowires or nanorods. The catalysis used TBHP as the oxygen source also obtained better catalytic effect. Chimentao *et al.*^20^ loaded silver onto magnetite and prepared Ag–Fe_3_O_4_ composite nanomaterials. They also used *tert*-butyl hydroperoxide as the oxygen source. When toluene was used as the solvent, Ag–Fe_3_O_4_ showed high catalytic activity and is very easy to separate and recover from the reaction system because of its magnetic properties. Silver has a high oxygen-binding affinity, allowing it to activate molecular oxygen and form reactive oxygen species, such as O^2−^ and O_2_^2−^. In addition, silver-based catalysts are known for their high selectivity for the epoxidation reaction, which is important to produce high-quality products. The high selectivity of silver-based catalysts can be attributed to the nature of the reactive oxygen species formed during the reaction, which are highly selective towards epoxide formation. In related reports, catalysts loaded with silver nanoparticles are very effective in styrene epoxidation reactions. Therefore, the application of silver catalysts in this field has attracted the interest of more and more researchers. However, Ag (0) is prone to agglomeration when present alone and has extremely poor stability, which seriously hinders its application. Ag(i) cannot be applied independently, so a carrier material or stabilizer is needed to overcome this shortcoming and achieve long-term application of Ag (0) and Ag(i).

Zeolite are widely used as carrier materials due to their 3D pore structure (large specific surface area) and stable framework structure (chemical, mechanical and thermal stability). Therefore, silver-based zeolites have attracted the attention and research of many scientific researchers, and have achieved rapid development. Zeolite are an effective support for silver-based catalysts, as they provide a high surface area and a controlled pore size distribution. Among the various types of zeolites, 4 Å zeolite have been shown to be effective for a range of reactions, including the epoxidation of olefins. There are some common methods for loading metals on a carrier such as deposition method, precipitation method, impregnation method, gas-phase deposition method, ion exchange method, hydrothermal method, plasma spraying method, electrodeposition method, and so on. However, these methods almost always load silver on the surface of the carrier, which seriously affects the stability and reusability of the catalyst. In recent years, due to the wider surface area, huge pore size, crystalline nature, high porosities and great chemical and thermal stability, MOFs have become popular.^21^ However, the synthesis of MOFs has been affected by several exterior factors, such as the selection of organic ligands, metal ion salts, molar ratio, pH, solvents and temperature of the reaction media.

In this study, a novel Ag-loaded 4 Å zeolite was prepared through the combined action of strong ultrasound and a high-voltage electrostatic field, which aimed to synthesize and evaluate silver-loaded 4 Å zeolite as highly effective catalysts for the epoxidation of styrene. This silver-based catalyst was highly attractive for the epoxidation of styrene due to its high activity, selectivity and ability to activate molecular oxygen, and performance of this silver-based catalysts for the epoxidation of styrene was further improve and the stability and reusability of the catalyst were enhanced. A new more stable and reusable method of loading metal was proposed.

## Experimental

### Materials

All chemicals were purchased from Sinopharm Chemical Reagent Co. Ltd and used as received without further purification.

### Preparation

Na_2_SiO_3_·9H_2_O (10.650 g) was added to a beaker and dissolved in distilled water (20 mL) with magnetic stirring until a clear solution formed. NaAlO_3_ (3.850 g) was added to another beaker and dissolved in distilled water (17.5 mL) with magnetic stirring until it formed a clear solution. Then, the clear solution was transferred to an ultrasonic bath and the NaAlO_3_ solution was added dropwise into the Na_2_SiO_3_ solution to form a colloidal solution with ultrasonic (50 Hz) and mechanical stirring for 1 h. Then the prepared silver diiodide complex ion solution, in which AgNO_3_ was respectively 0.073 g, 0.146 g, 0.219 g, 0.292 g and 0.365 g (the mass ratios were respectively 0.5, 1, 1.5, 2 and 2.5 wt% of the mass of zeolite, and named in short Z-Ag0.5, Z-Ag1, Z-Ag1.5, Z-Ag2 and Z-Ag2.5, respectively) was mixed with 15 mL distilled water, was added dropwise into the colloidal solution under ultrasonic conditions and kept it for 20 minutes. Then the colloidal solution was placed in high-voltage DC vertical electrostatic fields with electric field strengths of 25 kV (25 kV was the maximum voltage of the electrostatic field, because, if this value was exceeded, the sample in the electrostatic field was discharged, and the electrostatic field was broken down) with a distance between the positive and negative copper plates of 7 cm, in which the liquid level was 2 cm from the upper plate, for 1 h. The resulting mixtures were aged for 24 h, and then subjected to hydrothermal synthesis at 120 °C for 24 h after a certain amount of ethanol (the volume ratio of colloidal solution to ethanol is 5 : 1) was added into, cooled, the excess KI was added, and washed with distilled water (4–5 times), suction filtered and dried at 120 °C to give samples of 4 Å zeolite loaded diiodide complex ion. Then the sample was reduced with hydrazine hydrate, washed with distilled water (2–3 times), suction filtered and dried at 120 °C and finally obtained new samples of 4 Å zeolite loaded silver.

### Characterization

Elemental content of the sample was tested by Inductively Coupled Plasma Optical Emission Spectrometry (ICP-OES) using Agilent ICP-OES 725 ES (Agilent, US).

The morphology and structure of the prepared samples were characterized by scanning electron microscopy (SEM) using a Phenom machine (Phenom Pro, Netherlands).

The phase structure was investigated by powder X-ray diffraction patterns with a 2*θ* range of 5–60° (XRD, D/max 2500 PC, Rigaku, Japan).

Pyrolysis analysis was carried out by Simultaneous Thermal Analysis (STA, Netzsch, STA449F3).

**Scheme 1:**
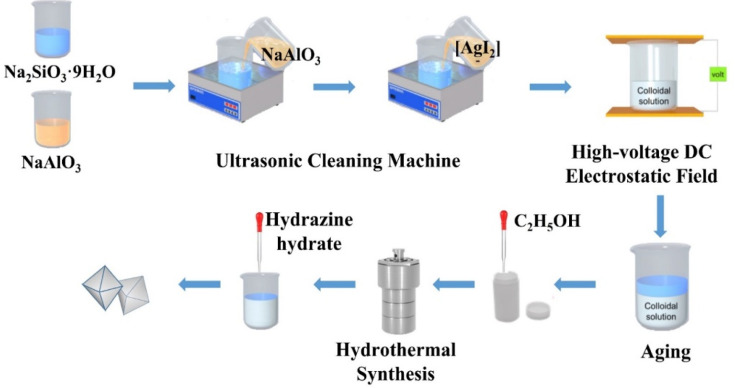
The synthesis of octahedral 4 Å zeolite loaded Ag.

The pore size distribution and the BET specific surface area were determined by the BJH (Barrett–Joyner–Halenda) and BET (Brunauer, Emmett, and Teller) methods, respectively. The temperature program for the degassing procedure consisted of pretreating at 70 °C for 15 min, heating to 120 °C at 10 °C min^−1^, holding the temperature at 120 °C for 15 min, then heating to 300 °C and holding for 3 h (Scheme 1).

### Catalytic performance

**Scheme 2:**
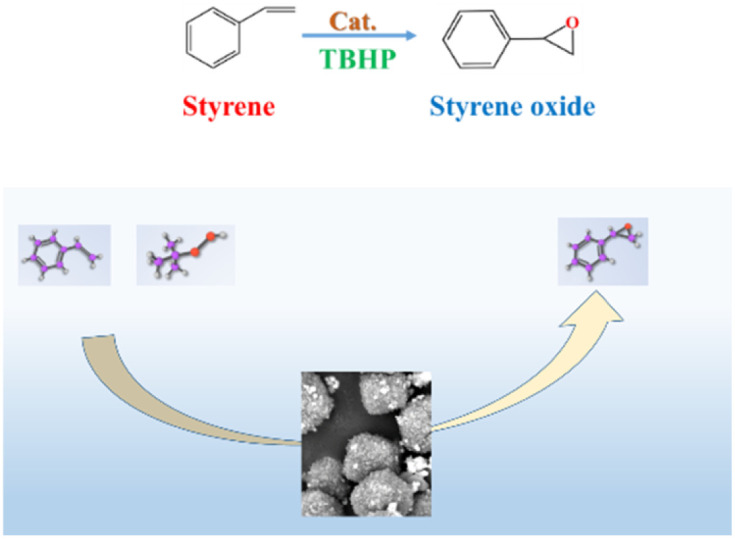
The oxidation reaction of styrene catalyzed by octahedral 4 Å zeolites loaded Ag.

The octahedral 4 Å zeolites loaded Ag combined action of strong ultrasound and a high-voltage electrostatic field were applied as catalysts for the epoxidation of styrene as follows: The octahedral 4 Å zeolites loaded Ag, Styrene (1 mL, is about 8.2 mmol at the room temperature), oxidant (*tert*-butyl hydro peroxide, 5 mL) and solvent (acetonitrile, 5 mL) were charged into a test tube fitted with a magnetic stirrer and condenser (Scheme 2). Reactions were carried out at 82 °C^22,23^ for 8 h under a N_2_ atmosphere. The solid catalyst was recovered by centrifugation, and the solution was filtered through a syringe filter. Product analysis from styrene oxidation experiments was achieved by gas chromatography (GC) (Shimadzu 2010 Plus, RTX-5 capillary column, flame ionization detector), and GC-MS (Agilent 5975C). The GC was performed with a temperature ramp of 10 °C min^−1^ from 60 °C to 280 °C and kept at 280 °C for 2 min. The conversion of styrene and the content of each product were calculated from the GC peak areas. GC analysis results for styrene epoxidation are in Fig. S1,† which shows that they correspond to *tert*-butyl hydro peroxide, styrene, benzaldehyde, phenylacetaldehyde and styrene oxide at 2.53, 4.40, 5.48, 6.80 and 7.22 minutes respectively, being basically the same as the GC spectra in the instrument database. In Fig. S2,† it is shown that GC analysis results for styrene epoxidation under optimal conditions and the reaction obtained the target product best.

### Mechanism analysis

**Scheme 3:**
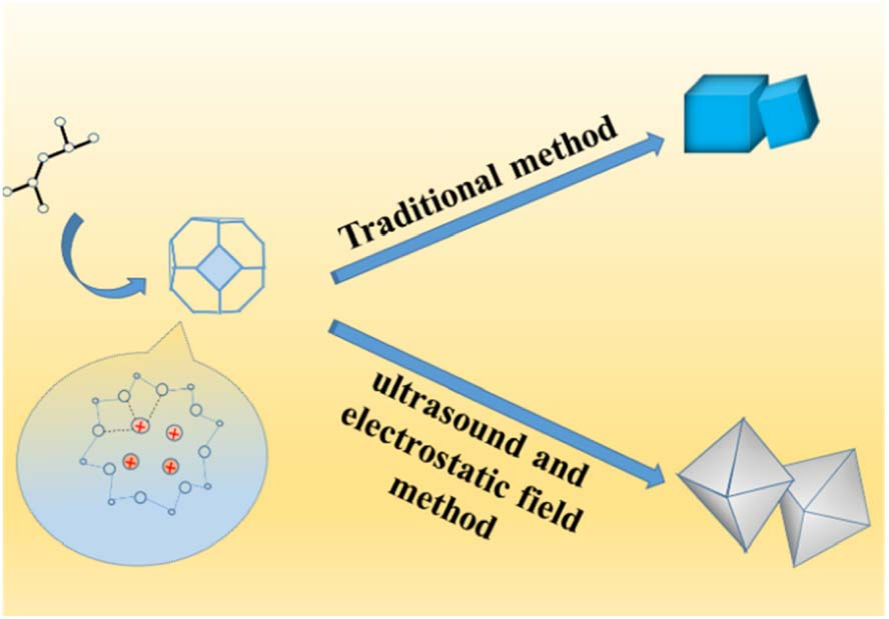
The formation of octahedral 4 Å zeolites loaded Ag.

In the structure of traditional 4 Å zeolite (as LTA), cations that balance the negative charge of the aluminosilicate zeolite framework is located in the pores and cages of the zeolite.^24–31^ In the 8-membered ring of LTA zeolite, each Na^+^ can be close to skeleton oxygen atoms, and may jump disorderly or stand still at equivalent positions, but occupy different positions in the 8-membered ring.^32^ Because SiO_2_ is an excellent insulator with little charge leakage and almost no interface state, the external electric field has almost no influence on it under normal circumstances.^33,34^ However, when the thickness of the SiO_2_ layer is close to or less than 1 nm, the electric field effect becomes large.^35–38^ In this work, strong ultrasonic cavitation^39–41^ made the particles more uniform and the thickness of colloidal SiO_2_ thinner to significantly less than 1 nm, which made the vertical electric field effect have a significant impact on the system. Firstly, the anions and cations in the 4 Å zeolite (as LTA) framework as well as silver diiodide complex ion were strongly polarized. In addition, because the entire system was colloid in the process of synthesizing zeolite, the colloid itself was also affected by the vertical electric field effect.^42,43^ In addition, because the system was a colloidal aqueous solution, the colloid settled due to gravity when it was left standing, forming an interface with the aqueous solution. Under the action of strong vertical electric field force and gravity, the surface tension of water and the colloid increased, and the electric dipole also increased. In the aging process, water and colloids strongly polarized by the vertical electric field still had a strong electric dipole moment.^44,45^ As a result, in the initial stage of the formation of zeolite nuclei, when the electrostatic field voltage was greater than or equal to 25 kV, the negatively charged central oxygen atom on the original framework and the free Na^+^ nearby^46^ were separated and silver diiodide complex ion might take the place some of the negatively charged central oxygen atom through the combined action of electric field force, surface tension and electric dipole moment until the original skeleton was stretched as a whole until the morphology of the material became octahedral (shown in Scheme 3). Under the action of a strong electric field, only did the morphology of the material change and become an octahedron, and this octahedral structure was more mesoporous, but the crystal structure itself did not fundamentally change yet, which can be seen in the XRD patterns (shown in Fig. 5), so an octahedron with mesoporous material which loaded [AgI_2_]^−^ was formed.^32,47–52^ After aging and hydrothermal synthesis, the zeolite framework was finally formed with [AgI_2_]^−^. Ethanol was added before hydrothermal synthesis in order to place ethanol into the structure to increase the pore structure and more silver atoms in the silver diiodide ions were exposed to facilitate subsequent *in situ* reduction before which the excess KI was added in order to remove AgI. Because the original skeleton were stretched as a whole to make the morphology of the material become octahedral which structure had more mesoporous though the crystal structure itself did not fundamentally change yet, the pore volume and pore channel increase^53–56^ as well as Ag evenly dispersed inside the octahedral 4 Å zeolite structure, which explains that the silicon-to-aluminum ratio and XRD diffraction peaks with the peaks of Ag were completely consistent with traditional 4 Å zeolite, but BET and BJH analysis showed formation of a mesoporous material rather than clogging the pores due to surface-loaded silver and the pores become smaller, and the particle size increased in SEM images, improving the catalytic ability of the catalyst.

## Results and discussion

Due to the cations that balance the negative charge of the aluminosilicate zeolite framework being located in the pores and cages of the zeolite within the framework structure,^29–34^ and the positions of the cations changing when they are affected by the energy of the surrounding environment, so energy can help the synthesis and improvement of zeolites.^32^ The effect of external energy fields such as microwaves^30^ and electrostatic field^31^ may also help to change the spatial structure of some zeolites and break some chemical bonds or activate zeolites. After many experiments and tests, it was found that silver could first be evenly dispersed inside the octahedral 4 Å zeolite in the form of complex ions, and then underwent *in situ* reduction through experimental means. Finally, a silver-supported zeolite catalyst with good stability and excellent catalytic performance was obtained.

The novel silver-loaded octahedral 4 Å zeolite synthesized by the combined action of strong ultrasound and a high-voltage electrostatic field were characterized using several analytical techniques to determine their physicochemical properties. The characterization studies included:

Firstly, Z-Ag0.5, Z-Ag1, Z-Ag1.5, Z-Ag2 and Z-Ag2.5 were respectively conducted ICP-OES and BET tests to test the basic properties of catalysts loaded with different amounts of silver.

### ICP-OES analysis

**Fig. 1:**
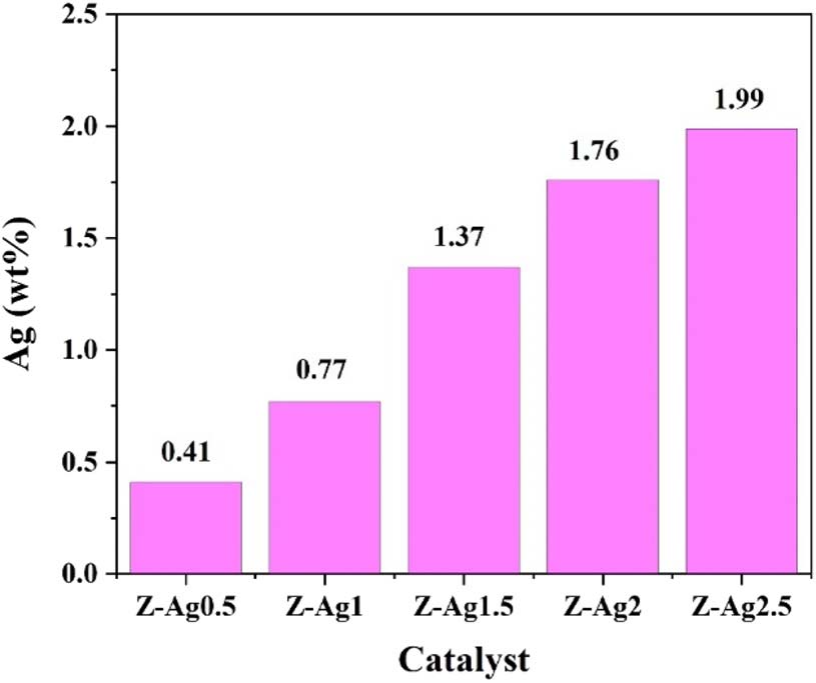
Silver contents of Z-Ag0.5, Z-Ag1, Z-Ag1.5, Z-Ag2 and Z-Ag2.5 determined by ICP-OES.

Silver content in five samples determined by ICP-OES (in Table 1). In Fig. 1, it is seen that the silver content gradually increases from Z-Ag0.5 to Z-Ag2.5, which shows that silver was successfully loaded into the zeolite through the combined method of strong ultrasound and a high-voltage electrostatic field. It also shows that the amount of silver loaded on the zeolite also increased as the amount of the silver diiodo complex ions added increased, being basically linear.

**Table 1:** The test data on Ag in ICP-OES

Sample	Sample quality *m*_0_ (g)	Constant volume *V*_0_ (mL)	Element	Concentration of tested element in the solution *C*_0_ (mg L^−1^)	Content of Ag (wt%)
Z-Ag0.5	0.0736	50	Ag	6.01	0.41
Z-Ag1	0.0668	50	Ag	10.26	0.77
Z-Ag1.5	0.0634	50	Ag	17.33	1.37
Z-Ag2	0.0836	50	Ag	29.49	1.76
Z-Ag2.5	0.0792	50	Ag	31.48	1.99

### BET analysis

**Fig. 2:**
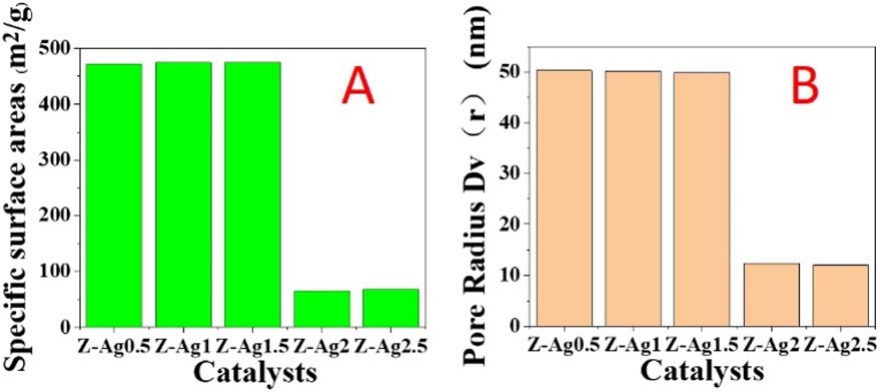
Specific surface areas (A) and pore diameters (B) of Z-Ag0.5, Z-Ag1, Z-Ag1.5, Z-Ag2 and Z-Ag2.5 catalysts.

In Fig. 2, it is seen that the specific surface area and pore diameter of samples of Z-Ag0.5, Z-Ag1 and Z-Ag1.5 are large and very close, but those of Z-Ag2 and Z-Ag2.5 drop sharply, and they are very close. This shows that silver was very uniformly dispersed inside the zeolite and did not occupy the pores of the zeolite in the three samples of Z-Ag0.5, Z-Ag1 and Z-Ag1.5. When more diiodosilver complex ions were added, they occupied the entire pore space, causing the specific surface area and pore size to decrease sharply, which prevented the internal silver from contacting the reactants, greatly reducing its catalytic activity, which is consistent with the subsequent reaction results.

### Catalytic performance

**Fig. 3:**
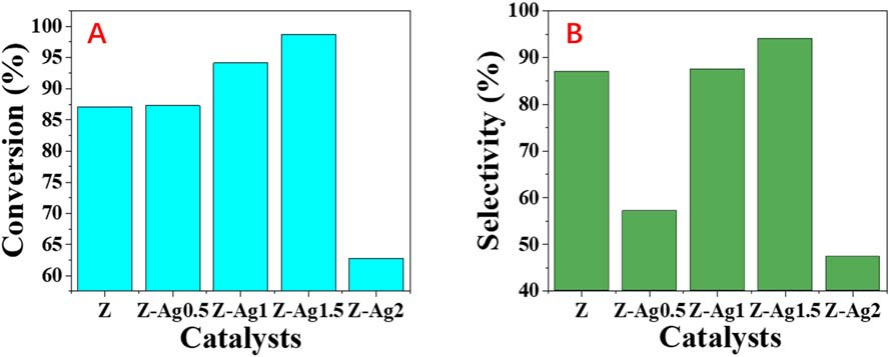
Styrene conversions (A) and styrene oxide selectivities (B) obtained over Z, Z-Ag0.5, Z-Ag1, Z-Ag1.5, Z-Ag2 and Z-Ag2.5 catalysts.

GC analysis results for styrene epoxidation using the catalysts of Z-Ag0.5, Z-Ag1, Z-Ag1.5, Z-Ag2 and Z-Ag2.5 are shown in Fig. 3. In Fig. 3, it is seen that the conversion and selectivity of the catalytic reaction from samples Z (the zeolite without Ag) to Z-Ag1.5 gradually increase with the increase of silver loading, while the conversion and selectivity of Z-Ag2 and Z-Ag2.5 decreased, which is consistent with the previous BET analysis that the specific surface area and pore size decreased leading to reduce the contact between silver and reactants.

Then, due to the silver contents of Z-Ag1.5 was very high and its specific surface area and pore diameter were the best among Z-Ag0.5, 1, 1.5, 2 and 2.5, Z-Ag1.5 was used to test.

### BET and BJH analysis

**Fig. 4:**
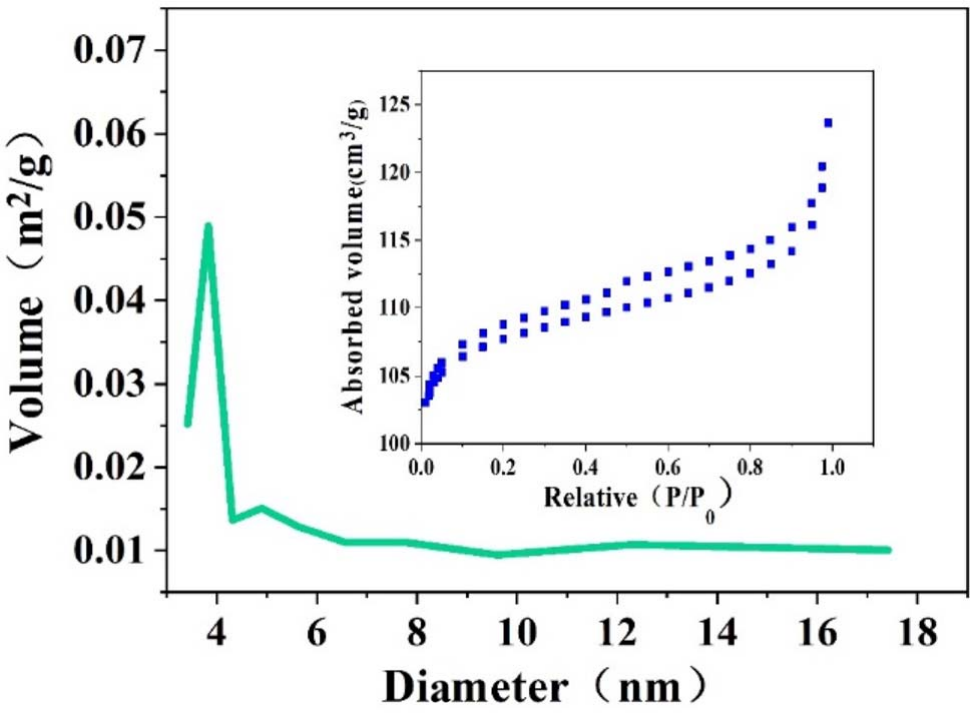
N_2_ adsorption–desorption isotherm and the pore size distribution of Z-Ag1.5.

The pore size distribution diagram and N_2_ adsorption–desorption isotherm of Z-Ag1.5 are shown in Fig. 4. According to the classification of the International Union of Pure and Applied Chemistry (IUPAC), it can be seen that the isotherm is type IV pattern and the closing points of the hysteresis loop are at the start and end points, indicating that the pores of the zeolite are well connected and have a uniform mesoporous structure (seen in Fig. 4). In the pore size distribution diagram of the material, it can be seen that it is mesoporous material. It can be concluded that the octahedral 4 Å zeolite was a mesoporous material through the combined action of strong ultrasound and a high-voltage electrostatic field.

BET calculations showed the specific surface areas, pore volume and pore radius of the samples of the octahedral 4 Å zeolite through the combined action of strong ultrasound and a high-voltage electrostatic field (the 4 Å zeolite-UE in short), the Z-Ag1.5 and the octahedral 4 Å zeolite loaded the same amount of silver by the impregnation method (the Z-Ag-IM in short) in Table 1. This shows that although the new octahedral 4 Å zeolite is loaded with silver, the specific surface area and pore size do not change much compared with the octahedral 4 Å zeolite through the combined action of strong ultrasound and a high-voltage electrostatic field while those of the octahedral 4 Å zeolite loaded silver by the impregnation method change significantly, which further illustrates that the loaded metallic silver was not simply loaded on the surface of the octahedral 4 Å zeolite, but evenly dispersed inside the octahedral 4 Å zeolite structure (Table 2).

**Table 2:** The results of BET of the 4 Å zeolite-UE, the Z-Ag1.5-UE and the Z-Ag1.5-IM

Catalyst	Surface area (m^2^ g^−1^)	Pore radius Dv (*r*) (nm)
4 Å zeolite-UE	468.72	47.82
Z-Ag1.5-UE	474.39	49.71
Z-Ag1.5-IM	15.28	4.74

### X-ray diffraction (XRD) analysis

**Fig. 5:**
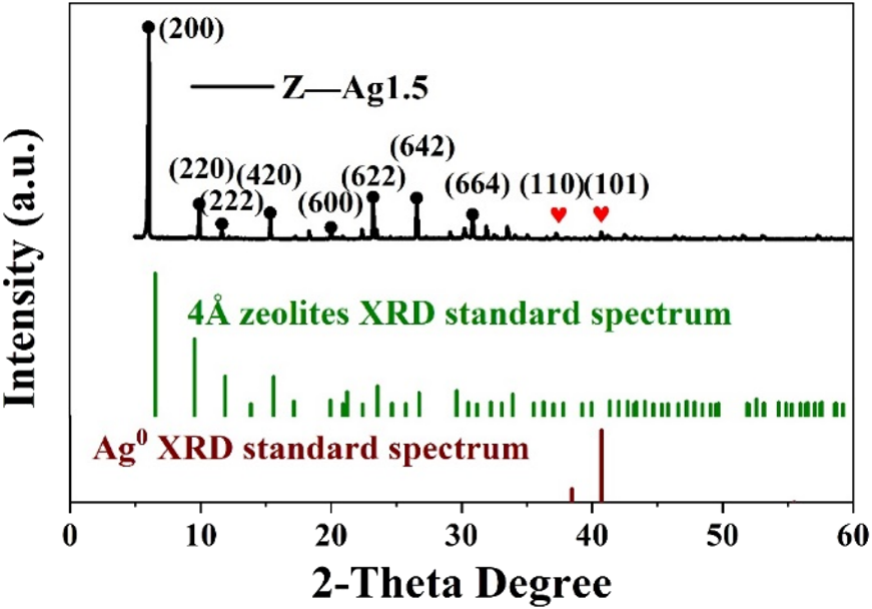
XRD patterns of Z-Ag1.5 and the 4 Å zeolite XRD and Ag0 standard patterns.

The crystal structure of the catalyst was determined using XRD. In Fig. 5, it is shown that the XRD patterns with clear reflections are at 2*θ* of 7.2, 10.2, 12.4, 16.1, 21.6, 24.0, 27.1, 29.9 and 34.1°. These diffraction peaks correspond to the crystal planes of (200), (220), (222), (420), (600), (622), (642), (644) and (664) of the 4 Å zeolite (JCPDS: No. 97-020-1472), respectively. And there are two peaks at 38.4 and 40.7°, which correspond to the crystal planes of (110) and (101) of Ag0 (JCPDS: No. 97-067-1056), respectively. The XRD pattern of the synthesized silver-loaded octahedral 4 Å zeolite showed a typical 4 Å zeolite crystal structure, indicating that the silver loaded through the combined action of strong ultrasound and a high-voltage electrostatic field did not alter the crystal structure of the octahedral 4 Å zeolite. The XRD analysis revealed that the synthesized catalysts had a pure crystalline structure of octahedral 4 Å zeolite with additional peaks corresponding to the presence of Ag^0^.

### SEM analysis

**Fig. 6:**
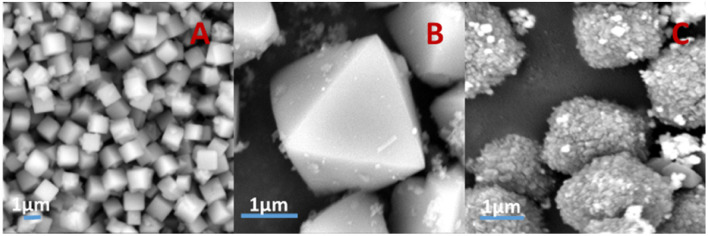
SEM images of traditional 4 Å zeolites (A), and silver-loaded octahedral 4 Å zeolite before reduction (B) and after reduction (C).

Fig. 6 shows SEM images of the silver-carrying zeolite. It can be seen that the traditional 4 Å zeolites (in A) and the silver-loaded octahedral 4 Å zeolite before reduction (in B) is regular and smooth-faced and the silver-loaded octahedral 4 Å zeolite after reduction (in C) is regular and scaly surface. After the action of a strong external field, the 4 Å zeolite deformed and the silver diiodo ions were evenly dispersed within the 4 Å zeolite structure. After reduction, the morphology of octahedral 4 Å zeolites has big change, which exposed more silver and also increased the specific surface area, improving the catalytic performance of the catalyst. This shows that although the new molecular sieve is loaded with silver, the specific surface area and pore size do not change much. This further illustrates that the loaded metallic silver was not simply loaded on the surface of the 4 Å zeolite, but evenly dispersed inside the octahedral 4 Å zeolite structure.

### XPS analysis

**Fig. 7:**
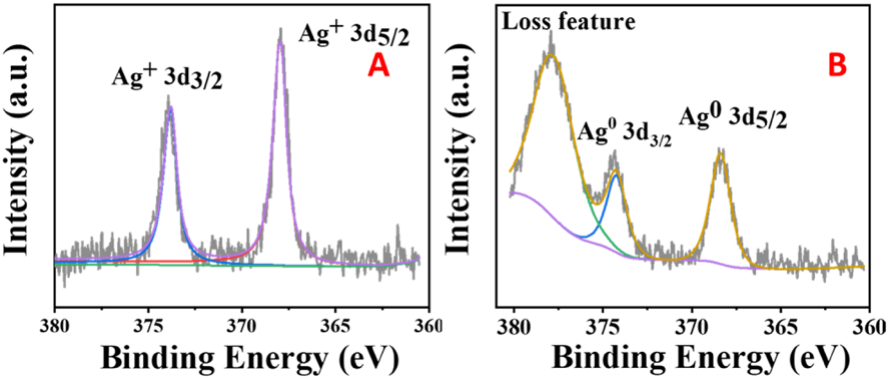
XPS images of silver-loaded octahedral 4 Å zeolite before reduction (A) and after reduction (B).

Fig. 7 shows XPS images of the silver-loaded zeolite before and after it was reduced. It can be seen that before reduction, silver still exists in a monovalent form (diiodosilver complex ion). After reduction, the monovalent silver is gone, and Ag^0^ appears.

### Zeta potential analysis

**Fig. 8:**
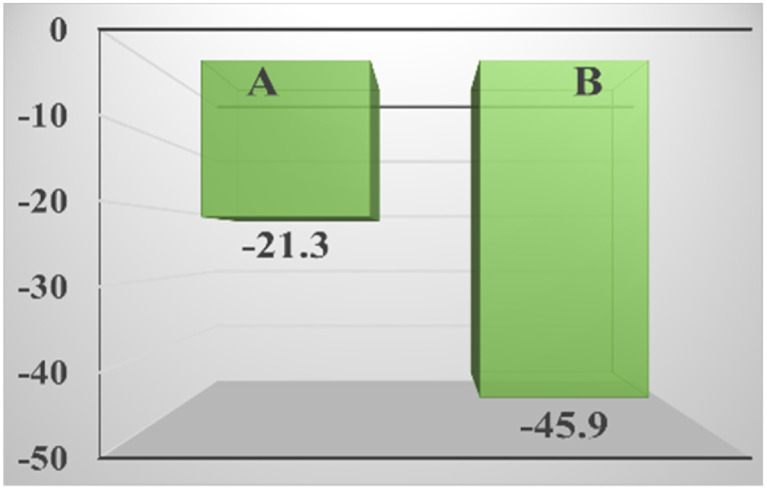
Zeta potential of the zeolite loaded with silver by the impregnation method (A) and the zeolite loaded with silver in the strong ultrasound and electric field (B).

It can be seen in Fig. 8 that compared with the zeolite loaded with silver by the impregnation method, the absolute value of the zeta potential of the zeolite loaded with silver in the strong ultrasound and electric field is larger, indicating that it has higher stability, and it is more electronegative, which is why it has better performance. Although the potential of styrene is also negative, the potential of TBHP is positive,^57^ which strongly attracts styrene and the catalyst, not only allowing the reaction to proceed smoothly, but also allowed the catalyst to fully contact the reactants and exert its catalytic effect.

### TG analysis

**Fig. 9:**
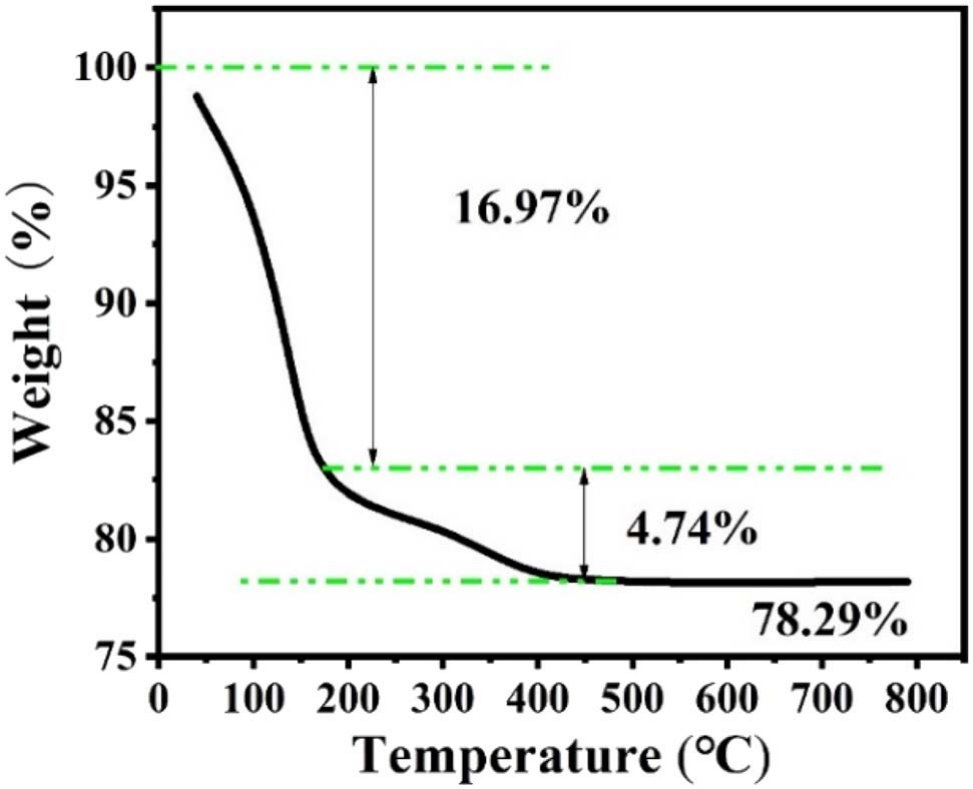
The thermogravimetric analysis of Z-Ag1.5.

**Fig. 10:**
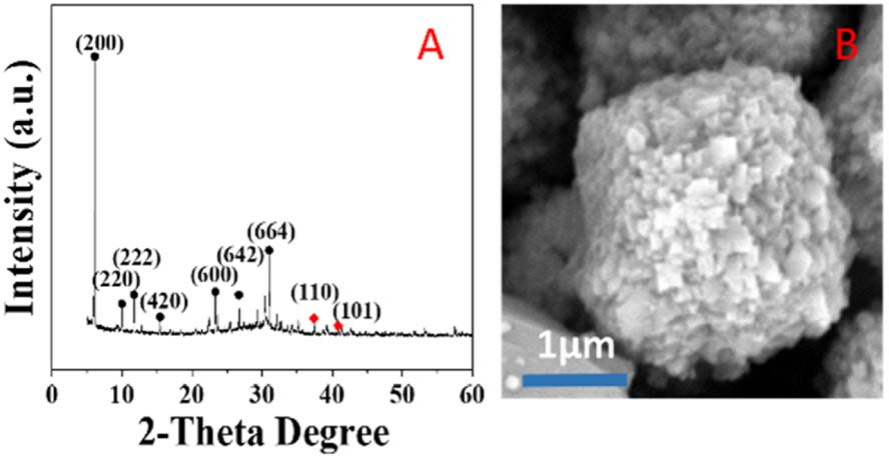
XRD pattern (A) and SEM image (B) of the Z-Ag1.5 after the thermogravimetric analysis.

Fig. 9 shows the thermogravimetric analysis under nitrogen of the Z-Ag1.5. Analysis of the weight loss curve shows that the zeolite has a thermal weight loss ratio of 16.97% in the range of 0 to 175 °C, due to the volatilization of water molecules adsorbed on the outer surface of the zeolite. The thermal weight loss ratio of 4.74% in the range of 175 °C to 500 °C is due to a surface transformation of the surface species.^58^ After 500 °C, there is almost no weight loss, and the crystal morphology and the peaks of XRD patterns hardly change after the thermogravimetric analysis at 800 °C as shown in Fig. 10, which shows that the high temperature thermal stability of the sample was excellent, overcoming the shortcomings of poor thermal stability generally associated with mesoporous zeolites and facile agglomeration of silver at high temperatures.

### Catalytic performance

**Fig. 11:**
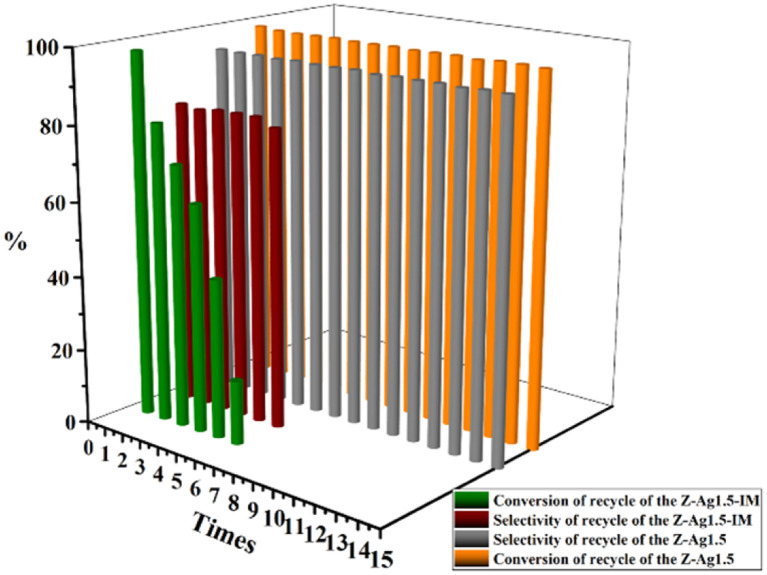
Selectivity and conversion of recycle of the Z-Ag1.5-IM and the Z-Ag1.5.

**Fig. 12:**
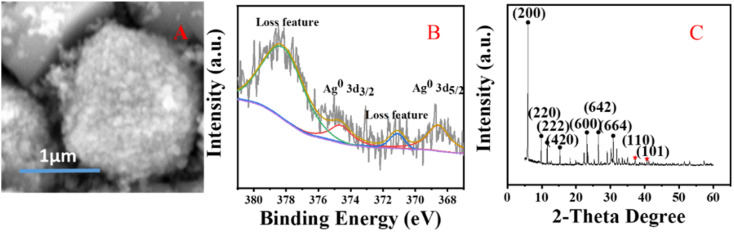
SEM (A) and XPS images (B) and XRD pattern (C) of the Z-Ag1.5 after fifteen times recycled.

GC analysis results for styrene epoxidation using the catalysts of Z-Ag1.5, investigated by varying the reaction conditions with the amount of catalyst, temperature, and reaction time (in Table 3). It can be seen in Table 3 that the selectivity of the reaction increases as the amount of catalyst increases. However, due to the formation of by-products, longer or shorter reaction time and higher or lower reaction temperature will increase the formation of by-products and reduce the selectivity. Therefore, there is an optimal condition for the selectivity of the reaction, which is marked red in the table, being 20 mg of catalyst, 82 °C^22,23,37–40^ and 6 h reaction time. GC analysis results for styrene epoxidation using the catalysts of Z-Ag1.5-IM and Z-Ag1.5 are shown in Fig. 11. Due to using Z-Ag1.5, the conversions and selectivity of styrene greatly improved, reaching a maximum of 98% and 94%, while those are of 96% and 82% using Z-Ag1.5-IM as well as 42% and 87% using the zeolite without Ag. And its TOFs are very high, with the highest value reaching about 500 h^−1^, which is much higher than the 28 and 26 h^−1^ in relevant literature.^18,23^ In Fig. 11, the conversion rate and selectivity of the reaction were very high when the catalyst was used at the first time, which is mainly due to the catalytic activity of octahedral 4 Å zeolite loaded Ag by the impregnation method is very good. But the conversions dropped sharply in being recycled, and almost no activity after six uses, which is mainly due to the Ag impregnated on the zeolite surface had poor stability leading to easily shed. However, after the Z-Ag1.5 was recycled more than fifteen times, the conversions and selectivity of styrene remained unchanged, the crystal morphology, the peaks of valence of Ag and XRD patterns also hardly changed (in Fig. 12), and the mass percentage of Ag had hardly changed and was still about 3.17 wt% after determined by ICP-OES. On the other hand, combined with the BET and BJH data and SEM images, this indicates that when using Z-Ag1.5, due to Ag was very evenly dispersed inside the octahedral 4 Å zeolite structure, the catalyst was very stable, which catalytic activity is very high and cycleability is very good.

**Table 3:** Epoxidation of styrene under various reaction conditions^a^

Entry	Amount of catalyst (g)	Temperature (°C)	Time (h)	Conversion (%)	Selectivity (%)	TOF (h^−1^)
1	0	82	6	19.6	81.23	—
2	0.01	82	6	56.23	93.21	564.67
3	0.02	82	6	98.24	94.35	499.31
4	0.03	82	6	98.32	94.50	333.67
5	0.02	70	6	60.43	36.33	118.26
6	0.02	90	6	97.02	82.58	431.59
7	0.02	82	4	43.22	23.17	80.92
8	0.02	82	8	97.54	67.59	266.36
9	0.02	82	10	98.67	46.34	147.78

a *TOF = *n*(SO)/(*n*(Ag)*T), where *n*(SO) = *n*(Styrene) *Con.*Sel. = 8.2 mmol*Con.*Sel., *n*(Ag) = m(Cat.) *0.0137/108, and *T* is the reaction time.

## Conclusions

In conclusion, a high catalytic activity, selectivity, stability, and reusability of silver-loaded octahedral 4 Å zeolite was prepared through the combined action of strong ultrasound and a high-voltage electrostatic field, and it was demonstrated in the epoxidation of styrene in this study. Due to the silver nanoparticles were evenly dispersed inside the octahedral 4 Å zeolite structure, the strong interaction between them and the octahedral 4 Å zeolite structure played a crucial role in enhancing the catalytic activity and selectivity of the catalyst. After using this catalyst, the efficiency and the selectivity of the reaction were improved, and the stability of catalyst was good, which can lead to significant cost savings in the production process and pave the way for future research in the field of heterogeneous catalysis, and contribute to the advancement of green chemistry and the reduction of environmental impact.

## Data availability

The data underlying this article are available in the article and in its online ESI.†

## Author contributions

The manuscript was written through contributions of all authors. All authors have given approval to the final version of the manuscript. These authors contributed equally.

## Conflicts of interest

There are no conflicts to declare.

## Supplementary Material

RA-014-D4RA00758A-s001
