# Supplementary material for: A novel Ag-loaded 4 Å zeolite as an efficient catalyst for epoxidation of styrene
Source: RSC Adv. 2024 Jun 19;14(28):19735–43. doi: 10.1039/d4ra00758a (PMC11188665; doi:10.1039/d4ra00758a)
Supplement: RA-014-D4RA00758A-s001 [file RA-014-D4RA00758A-s001.pdf]

Electronic Supplementary Material (ESI) for RSC Advances.
This journal is © The Royal Society of Chemistry 2024

A novel Ag-loaded 4Å zeolite as an Efficient Catalyst for Epoxidation of Styrene

Junzhong Wang[a,b], Qiancheng Zhang[a,b], Ying Li[a,b], Tong Xu [a,b], Yinghui Sun*[a,b] and Jie Bai*[a,b]

a. College of Chemical Engineering, Inner Mongolia University of Technology, Hohhot, 010051, People's Republic of China

b. Inner Mongolia Key Laboratory of Industrial Catalysis, Hohhot, 010051, People's Republic of China

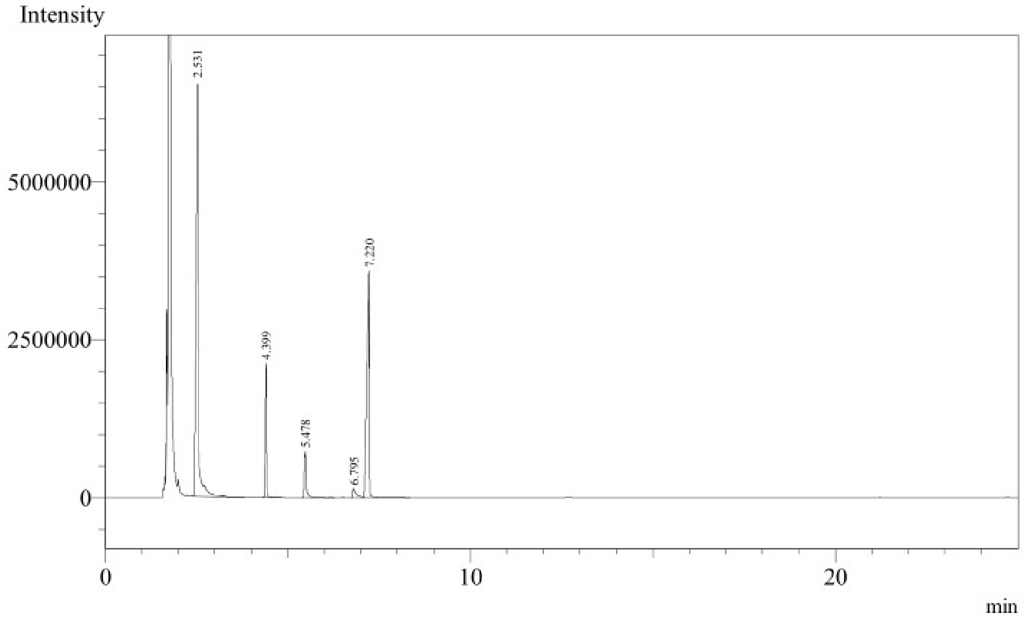

Figure S1. GC analysis results for styrene epoxidation

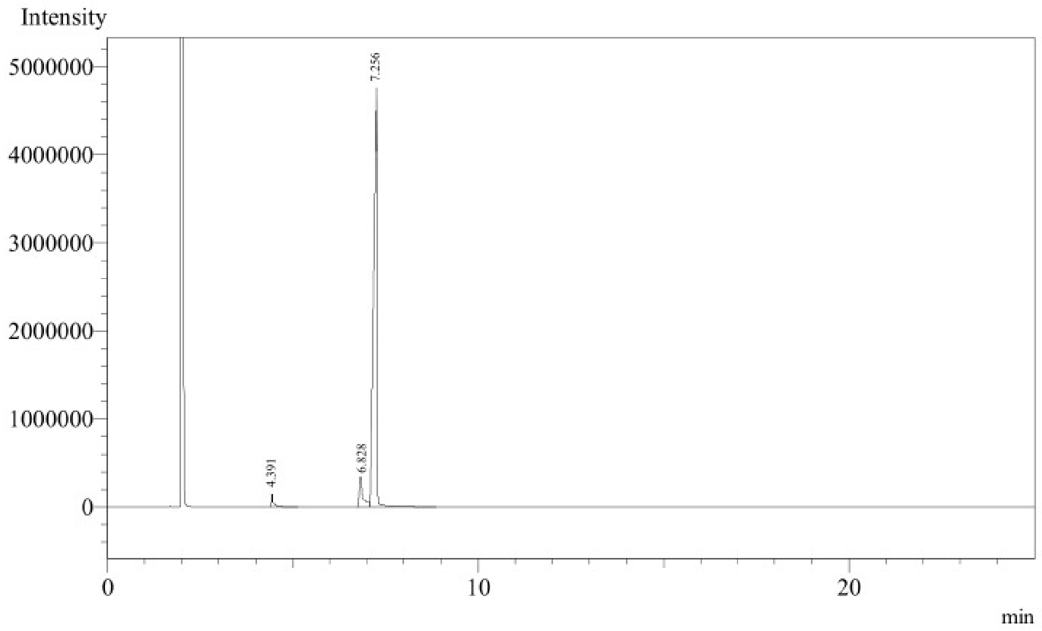

Figure S2. GC analysis results for styrene epoxidation under optimal conditions
